# Correction of Arterial-Phase Motion Artifacts in Gadoxetic Acid-Enhanced Liver MRI Using an Innovative Unsupervised Network

**DOI:** 10.3390/bioengineering10101192

**Published:** 2023-10-13

**Authors:** Feng Pan, Qianqian Fan, Han Xie, Chongxin Bai, Zhi Zhang, Hebing Chen, Lian Yang, Xin Zhou, Qingjia Bao, Chaoyang Liu

**Affiliations:** 1 Department of Radiology, Union Hospital, Tongji Medical College, Huazhong University of Science and Technology, Wuhan 430022, China; uh_fengpan@hust.edu.cn (F.P.); qianqianfan@hust.edu.cn (Q.F.); hebing1027898473@126.com (H.C.); yanglian@hust.edu.cn (L.Y.); 2 State Key Laboratory of Magnetic Resonance and Atomic and Molecular Physics, Innovation Academy for Precision Measurement Science and Technology, Chinese Academy of Sciences, Wuhan 430071, China; yingxhgo@gmail.com (H.X.); zhangzhi@apm.ac.cn (Z.Z.); xinzhou@apm.ac.cn (X.Z.); 3 School of Information Engineering, Wuhan University of Technology, Wuhan 430070, China; bcx907372720@foxmail.com; 4 University of Chinese Academy of Sciences, Beijing 100864, China; 5 Optics Valley Laboratory, Wuhan 430074, China

**Keywords:** artifacts, gadoxetic acid, unsupervised machine learning, magnetic resonance imaging

## Abstract

This study aims to propose and evaluate DR-CycleGAN, a disentangled unsupervised network by introducing a novel content-consistency loss, for removing arterial-phase motion artifacts in gadoxetic acid-enhanced liver MRI examinations. From June 2020 to July 2021, gadoxetic acid-enhanced liver MRI data were retrospectively collected in this center to establish training and testing datasets. Motion artifacts were semi-quantitatively assessed using a five-point Likert scale (1 = no artifact, 2 = mild, 3 = moderate, 4 = severe, and 5 = non-diagnostic) and quantitatively evaluated using the structural similarity index (SSIM) and peak signal-to-noise ratio (PSNR). The datasets comprised a training dataset (308 examinations, including 58 examinations with artifact grade = 1 and 250 examinations with artifact grade ≥ 2), a paired test dataset (320 examinations, including 160 examinations with artifact grade = 1 and paired 160 examinations with simulated motion artifacts of grade ≥ 2), and an unpaired test dataset (474 examinations with artifact grade ranging from 1 to 5). The performance of DR-CycleGAN was evaluated and compared with a state-of-the-art network, Cycle-MedGAN V2.0. As a result, in the paired test dataset, DR-CycleGAN demonstrated significantly higher SSIM and PSNR values and lower motion artifact grades compared to Cycle-MedGAN V2.0 (0.89 ± 0.07 vs. 0.84 ± 0.09, 32.88 ± 2.11 vs. 30.81 ± 2.64, and 2.7 ± 0.7 vs. 3.0 ± 0.9, respectively; *p* < 0.001 each). In the unpaired test dataset, DR-CycleGAN also exhibited a superior motion artifact correction performance, resulting in a significant decrease in motion artifact grades from 2.9 ± 1.3 to 2.0 ± 0.6 compared to Cycle-MedGAN V2.0 (to 2.4 ± 0.9, *p* < 0.001). In conclusion, DR-CycleGAN effectively reduces motion artifacts in the arterial phase images of gadoxetic acid-enhanced liver MRI examinations, offering the potential to enhance image quality.

## 1. Introduction

Gadoxetic acid is an effective liver-specific contrast agent for magnetic resonance imaging (MRI), which is widely utilized in the detection of small hepatocellular carcinoma (HCC) lesions [1,2,3,4,5,6,7]. To ensure the early diagnosis of small HCC, it is crucial to assess arterial-phase hyperenhancement (APHE) according to the current Li-RADS criteria [8,9]. However, numerous research findings have acknowledged the occurrence of side effects, such as acute transient dyspnea or transient severe motion (TSM), following the administration of Gadoxetic acid [10,11,12]. These side effects can specifically result in significant motion artifacts in arterial-phase images [10,11]. Previous studies have reported a high incidence (5–18%) of severe degradation in image quality during the arterial phase of gadoxetic acid-enhanced MR scans [13]. Consequently, the accurate evaluation of APHE presentation becomes challenging, potentially leading to the incorrect classification of liver nodules. Thus, developing an appropriate motion artifact correction algorithm becomes essential for enhancing the quality of arterial-phase images in gadoxetic acid-enhanced liver MRI.

Prospective techniques for motion artifact correction involve real-time adjustments to image acquisition, often utilizing the optical tracking of target markers or continuous navigator scans [14,15]. However, these prospective solutions face significant challenges when applied to liver MRI due to the complexity of tracking nonrigid motion and the resulting considerably longer scanning duration [14,15]. In contrast, retrospective motion correction methods offer a different approach by making adjustments to the *k*-space or image data post-acquisition, without the need for specific tracking devices or navigators [15]. Among these retrospective techniques, the data-driven autofocusing motion correction approach holds promise as it can be easily implemented across all scanners [15]. Unfortunately, this approach faces obstacles in the form of a poorly conditioned and nonconvex optimization problem [15].

With the rapid advancements in deep learning technologies, the potential of deep learning for MRI motion correction has been extensively demonstrated, yielding promising outcomes [16,17,18,19,20,21,22,23,24,25,26,27]. Deep learning has emerged as a powerful tool in the field of MRI motion correction, offering a solution to address convergence issues often associated with retrospective techniques, as mentioned previously [16,18,28]. Early deep-learning models heavily relied on paired motion-free images for supervised learning, despite their proficiency in artifact correction [16,17,18,29]. Consequently, the feasibility of these supervised approaches diminishes due to the inherent challenge of acquiring paired motion-free images in clinics, especially in the context of enhanced MRI scans.

Afterwards, one notable breakthrough is the introduction of the Cycle-Consistent General Adversarial Network (CycleGAN) [20]. CycleGAN represents a significant advancement in the realm of motion artifact correction for liver MRI examinations [20,29]. A pivotal innovation in motion artifact correction using CycleGAN is the introduction of a new non-adversarial loss named cycle-consistency loss [20]. This loss function plays a crucial role in preserving vital image information and mitigating the risk of information loss during the image translation process. It ensures that the translation process from the motion-corrupted domain to the motion-free domain and back remains consistent, thus bolstering the correction of motion artifacts [20,29]. As a result, CycleGAN alleviates the need for paired motion-free and motion-corrupted images in clinical settings [20,29]. Furthermore, certain unsupervised methods (e.g., Cycle-MedGAN, etc.), building upon the traditional CycleGAN framework, have been proposed that incorporate other new non-adversarial losses, demonstrating more promising results in motion artifact corrections [17,19,20,28]. However, despite the promise of these networks, the challenges posed by motion artifacts in liver MRI examinations remain a formidable hurdle. The motion artifacts often manifest in diverse and unpredictable ways, which may not be adequately addressed by the straightforward application of the traditional CycleGAN framework [19,26,28,30]. To tackle this challenge, researchers have introduced end-to-end disentangled unsupervised networks, such as DUNCAN, designed for training using unpaired data, enabling the flexible and simultaneous correction of a range of MRI motion artifacts [26]. Experimental results demonstrate that the method is effective in removing artifacts and retaining anatomical details in images [26]. Nevertheless, the current disentangled framework poses complexity concerns, featuring a total of four encoders that not only elevate computational demands, but also introduce training challenges [26].

Therefore, despite these advancements, there remains an ongoing need for innovative solutions that can offer enhanced robustness, efficiency, and effectiveness in addressing motion artifacts. Inspired by the advancements mentioned above, this study proposes the end-to-end Disentangled Representation-Learning Cycle-Consistent Generative Adversarial Network (DR-CycleGAN), which seeks to push the boundaries of motion artifact correction in gadoxetic acid-enhanced liver MRI examinations by applying a modified disentangled representation technique and an improvement of non-adversarial losses. This network is built upon three key assumptions: 1. motion-corrupted images consist of two distinct domains—the content domain (motion-free images) and the artifact domain (motion artifacts)—while motion-free images possess only the content domain; 2. motion-corrupted images can be disentangled into content and artifact domains through two separately trained encoders, enabling the generation of motion-free images by utilizing a trained generator specifically for the content domain; 3. The breath-holding failure causes motion artifacts always along the phase-encoding direction, meaning a novel content-consistency loss can be designed to calculate the content consistency between the translated image and the input image along the phase-encoding direction. In this study, we evaluate the performance of DR-CycleGAN in correcting motion artifacts in arterial-phase images obtained from gadoxetic acid-enhanced liver MRI examinations in patients.

## 2. Materials and Methods

### 2.1. The Proposed DR-CycleGAN Structure

The architecture of DR-CycleGAN is illustrated in Figure 1. During the training stage, DR-CycleGAN takes unpaired motion-free and motion-corrupted images as inputs. Motion-free images exclusively represent the content domain, while motion-corrupted images encompass both the content and artifact domains. Similar to Cycle-GAN, DR-CycleGAN performs the translation of motion-corrupted images to motion-free images without requiring aligned pairs [20]. However, due to the diverse characteristics of motion artifacts, single-cycle mapping generators may not be sufficient to generate an optimal distribution, as mentioned in the introduction [20]. To address this limitation, DR-CycleGAN introduces two encoders, two generators, and two discriminators, based on convolutional neural networks (Figure 1 and Supplementary S1–S3):

- Two Encoders: A conventional “content-feature extraction” encoder ($E_{c}$) and an extra “artifact-feature extraction” encoder ($E_{a}$). They can enhance the disentanglement of content features (C) and artifact features (A) in motion-corrupted images ($x_{c}$). By employing both encoders ($E_{a}$ and $E_{c}$), the content and artifact features (C and A) in motion-corrupted images ($x_{c}$) are separated.
- Two Generators: $G_{f}$ and $G_{c}$, which were introduced to specifically generate motion-free and motion-corrupted images, respectively. $G_{f}$ can generate “motion-free” images (${\hat{x}}_{f}$, $y_{f}^{rec}$, and $y_{f}^{cyc}$) reconstructed from content features (C), while $G_{c}$ can generate different “motion-corrupted” images (${\hat{x}}_{c}$, $y_{c}^{rec}$, and $y_{c}^{cyc}$) reconstructed from concatenated content and artifact features (C⨁A).
- Two discriminators: $D_{f}$ and $D_{c}$, which are employed to distinguish between reconstructed motion-free images (${\hat{x}}_{f}$) and real motion-free images ($y_{f}$), as well as between fake motion-corrupted images (${\hat{y}}_{c}$) and real motion-corrupted images ($x_{c}$).

The primary objective of DR-CycleGAN is to accurately disentangle the content and artifact domains. This means that regardless of the source of content or artifact features, the reconstructed “motion-free” or “motion-corrupted” images should not be distinguishable from or should closely resemble real ones. To achieve this goal, the training process involves considering all separated content features and various combinations of content and artifact features extracted from both original motion-free and motion-corrupted images as inputs for different generators. By doing so, the network can effectively learn the optimal disentanglement between the content and artifact domains. This, in turn, enables DR-CycleGAN to reconstruct “motion-free” and “motion-corrupted” images that are indistinguishable from real ones. Following the training phase, motion artifact correction for test datasets exclusively utilizes the “content-feature extraction” encoder ($E_{c}$) and the generator $G_{f}$ to obtain motion-corrected images in an end-to-end manner (Figure 1). For a more detailed explanation of the DR-CycleGAN structures, please refer to Supplementary S1. More network details about encoders, generators, and discriminators can be found in Supplementary S2 and S3. The code of DR-CycleGAN was released on GitHub: https://github.com/baoqingjia/DR-CycleGAN (accessed on 15 August 2023).

**Figure 1:**
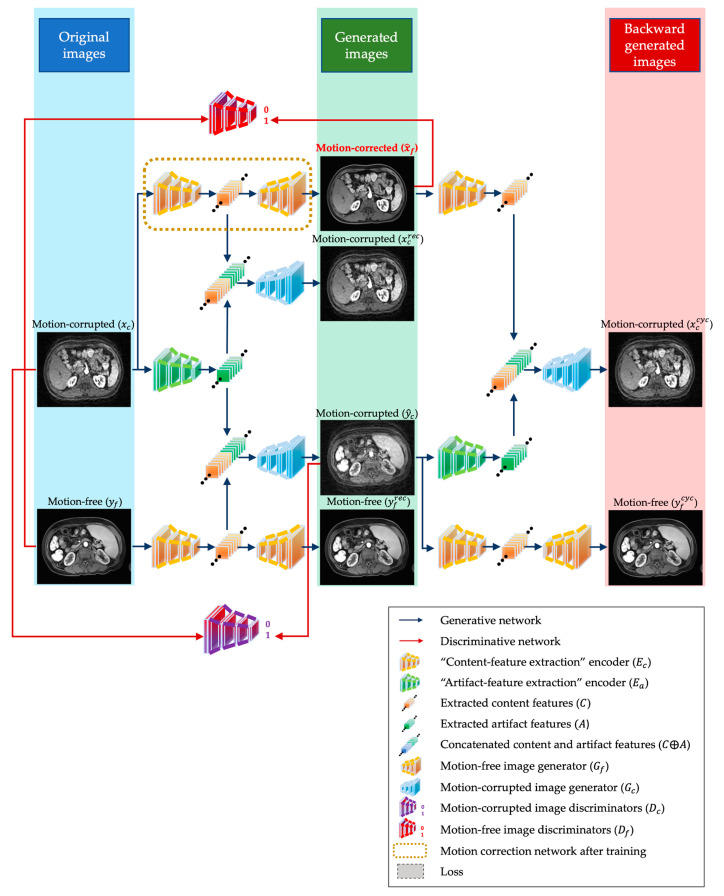
The structure illustration of the proposed DR-CycleGAN. Briefly, the proposed DR-CycleGAN for motion artifact correction has a content encoder ($E_{c}$) and an artifact encoder ($E_{a}$), two generators ($G_{c}$ and $G_{f}$), and two discriminators ($D_{f}$ and $D_{c}$). The network has six translation mappings between the motion-corrupted and motion-free image translation: $T_{c\to f}:x_{c}\to {\hat{x}}_{f}$, $T_{f\to c}:y_{f}\to {\hat{y}}_{c}$, $T_{c\to c}:x_{c}\to x_{c}^{rec}$, $T_{f\to f}:y_{f}\to y_{f}^{rec}$, $T_{f\to c}:{\hat{x}}_{f}\to x_{c}^{cyc}$, and $T_{c\to f}:{\hat{y}}_{c}\to y_{f}^{cyc}$, in which $x_{c}$, ${\hat{y}}_{c}$, $x_{c}^{rec}$, and $x_{c}^{cyc}$ are motion-corrupted images and $y_{f}$, ${\hat{x}}_{f}$, $y_{f}^{rec}$, and $y_{f}^{cyc}$ are motion-free images. Among these mappings, $T_{c\to f}:x_{c}\to {\hat{x}}_{f}$ can be used in motion artifact correction for test datasets after the training phase to obtain motion-corrected images. More explicit explanations can be found in Supplementary S1–S3. Abbreviations: T, translation mapping; c, motion-corrupted; f, motion-free; x, images sampled from motion-corrupted image dataset; y, images sampled from motion-free image dataset; ^, forward generated images; ${}^{rec}$, direct recovered images; ${}^{cyc}$, backward generated images; D, discriminator; E, encoder; C, content features; A, artifact features; G, generator.

### 2.2. Loss in the Training

As mentioned above, the training process of DR-CycleGAN strives to ensure that all reconstructed “motion-free” (represented by ${\hat{x}}_{f}$, $y_{f}^{rec}$, and $y_{f}^{cyc}$ in Figure 1 and Figure 2a) and “motion-corrupted” (represented by ${\hat{x}}_{c}$, $y_{c}^{rec}$, and $y_{c}^{cyc}$ in Figure 1 and Figure 2a) images, generated from various inputs, closely resemble real images. Therefore, it is crucial to establish a sound loss framework that encompasses comparisons between each type of reconstructed “motion-free” and “motion-corrupted” images with their original counterparts. Drawing from prior research, DR-CycleGAN incorporates three commonly used loss functions (Supplementary S2) [17,18,24,27,28]:

- Adversarial domain loss *(*$L_{adv}$*)*: it supervises the resemblance between motion-corrected images and the original motion-free images (${\hat{x}}_{f}$ vs. $y_{f}$), as well as the similarity between generated motion-corrupted images and the original motion-corrupted images (${\hat{y}}_{c}$ vs. $x_{c}$) (Figure 2a and Supplementary S2).
- Reconstruction loss *(*$L_{rec}$*)*: It is designed to minimize the pixel-wise difference between the input image and its reconstructed counterpart in the same domain translation ($x_{c}^{rec}$ vs. $x_{c}$ and $y_{f}^{rec}$ vs. $y_{f}$). Its primary objective is to ensure that eligible encoders and generators do not introduce any significant discrepancies during the reconstruction process (Figure 2a and Supplementary S2).
- Cycle-consistency loss *(*$L_{cycle}$*)*: it is another classic loss in CycleGAN and guarantees that the images generated backward closely resemble the originals ($x_{c}^{cyc}$ vs. $x_{c}$ and $y_{f}^{cyc}$ vs. $y_{f}$) (Figure 2a and Supplementary S2).

While the incorporation of these loss functions significantly enhances the training of DR-CycleGAN, enabling it to generate motion-corrected images that closely resemble the originals, it is essential to acknowledge an inherent limitation. These loss functions do not account for the potential correlation between paired motion-corrected/corrupted and original motion-corrupted/free images (${\hat{x}}_{f}$ vs. $x_{c}$ and ${\hat{y}}_{c}$ vs. $y_{f}$), which do not effectively prevent the introduction of spurious information during the cross-domain translation (or so-called “motion artifact correction”) process [31].

In reality, motion artifacts arising from breath-holding failures often exhibit a distinct characteristic—alignment along the phase-encoding direction [18]. This leads to noticeable discrepancies between motion-free and motion-corrupted images. Nevertheless, it is worth highlighting that despite the presence of motion artifacts caused by breath-holding failures, the overall total signal intensity along each column or direction remains relatively consistent between paired motion-free and motion-corrupted images. This consistency arises because the signals only undergo a shift along the phase-encoding direction without altering the total intensity in this specific direction [18]. Thus, the summation ratio of pixel values in every column along the phase-encoding direction tends to be remarkably consistent between paired motion-free and motion-corrupted images (Figure 2b). Inspired by this unique characteristic, we introduce a novel content-consistency loss function—$L_{sum}$. It harnesses the consistency in the summation ratios of pixel values in every column along the phase-encoding direction as a valuable constraint, contributing to the enhancement of motion artifact correction within DR-CycleGAN. The formula for $L_{sum}$ is as follows:

$$L_{sum}=\sum_{i=1}^{n}\left({\left\|\frac{{sum}_{i}(x_{c})}{{max}_{i}(x_{c})}-\frac{{sum}_{i}({\hat{x}}_{f})}{{max}_{i}({\hat{x}}_{f})}\right\|}_{1}+{\left\|\frac{{sum}_{i}(y_{f})}{{max}_{i}(y_{f})}-\frac{{sum}_{i}({\hat{y}}_{c})}{{max}_{i}({\hat{y}}_{c})}\right\|}_{1}\right)$$

where ${\left\|\cdot \right\|}_{1}$ denotes the L1-norm, n is the pixel number of the image column which equals 320, ${sum}_{i}$ is the summation of the pixel value in $i^{th}$ column, and ${max}_{i}$ is the maximum pixel value of the $i^{th}$ column. $L_{sum}$ serves to penalize content discrepancies between the original dataset images and their respective cross-domain translated counterparts (including $x_{c}$ vs. ${\hat{x}}_{f}$ and $y_{f}$ vs. ${\hat{y}}_{c}$). By including $L_{sum}$, content errors can be minimized after motion artifact correction, furthermore ensuring that the resulting motion-corrected images preserve anatomical details while preventing the introduction of spurious information.

In sum, the total loss function ($L_{total}$) of DR-CycleGAN comprises four components: $L_{adv}$, $L_{rec}$, $L_{cycle}$, and the proposed $L_{sum}$ (Figure 2a). The total loss can be expressed as follows:

$$L_{total}=L_{adv}+l_{1}L_{rec}+l_{2}L_{cycle}+{l_{3}L}_{sum}$$

where $l_{1}$, $l_{2},\;$and $l_{3}$ are the balance factors to ensure similar contributions among all these losses, which were set as 10, 10, and 0.5, respectively.

**Figure 2:**
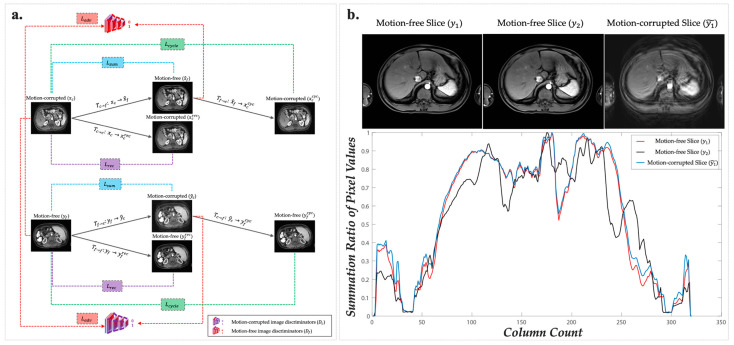
The structure illustration of applied loss in DR-CycleGAN. (**a**) Based on different reconstructed images obtained from six translation mappings, four types of loss were proposed to comprehensively compare different types of images: $L_{adv}$; $L_{rec}$; $L_{cycle}$; and $L_{sum}$. (**b**) Among four types of loss, $L_{sum}$ is a novel proposed content-consistency loss. After calculating the ratio of the summation of the pixel values along each column, the content information of $y_{1}$ (*red curve*) and ${\tilde{y}}_{1}$ (*blue curve*) at the same slice is much closer than image $y_{2}$ (*black curve*) at another slice. More explicit explanations can be found in Supplementary S1–S3. Abbreviations: T, translation mapping; c, motion-corrupted; f, motion-free; x, images sampled from motion-corrupted image dataset; y, images sampled from motion-free image dataset; ^, forward generated images; ${}^{rec}$, direct recovered images; ${}^{cyc}$, backward generated images; D, discriminator.

### 2.3. Motion Artifact Grading

For subjective evaluation, the severity of motion artifacts in arterial-phase MRI before and after motion correction was assessed using a classic five-point Likert scale: 1 = no artifact, 2 = mild artifacts, 3 = moderate artifacts, 4 = severe artifacts, and 5 = non-diagnostic (Figure 3) [10,12,18,32]. Two experienced radiologists (LY and FP) with 25 and 13 years of expertise in abdominal radiology, respectively, independently conducted semi-quantitative evaluations of motion artifact scales and other quantitative measurements. Inter-measurement agreement was determined using multi-measurement intraclass correlation coefficients (ICCs), categorized as poor (ICC, 0–0.40), moderate (ICC, 0.40–0.75), or good (ICC, >0.75) [32]. Any discrepancies in motion artifact scales were resolved through consensus after discussion between the two readers. Quantitative measurements were averaged and compared before and after motion correction. Drawing and measurement of regions of interest (ROIs) were performed on axial images using the MITK software (version v2021.10, https://www.mitk.org/wiki/The_Medical_Imaging_Interaction_Toolkit_(MITK), accessed on 12 October 2023), a free and open-source tool.

### 2.4. Training and Test Datasets

The retrospective collection of gadoxetic acid-enhanced liver MRI data for model training and testing was approved by the Institutional Review Board at Union Hospital, Tongji Medical College, Huazhong University of Science and Technology (No. 2020-336). The image data underwent deidentification preprocessing, and the need for informed consent from patients was waived. From June 2020 to September 2020, a total of 308 adults who underwent gadoxetic acid-enhanced liver MRI examinations were consecutively included from a single center (Union Hospital, Tongji Medical College, Huazhong University of Science and Technology) for model training (Supplementary S4) [33]. The MR image data (in DICOM file format with the same 320×320 resolution) were retrieved from the institutional digital system (Vue PACS, version 11.3.5.8902, Carestream Health, Concord, ON, Canada) and underwent deidentification preprocessing. Among these image data, 176 examinations (7045 slices) were acquired on a commercial 1.5T MR scanner (MAGNETOM Avanto, Siemens Healthineers, Erlangen, Germany), and another 132 examinations (9279 slices) were obtained on a 3T MR scanner (MAGNETOM Skyra, Siemens Healthineers, Germany). The scanning protocols followed standard procedures using 3-Dimensional Volumetric Interpolated Breath-hold Examination (3D-VIBE) sequences, as described in previous studies (Supplementary S5) [34]. Out of the 308 examinations, 58 (4005 slices) had no artifacts (grade-1), and the remaining 250 examinations (12,319 slices) had grade-2 to -5 motion artifacts, forming the training dataset (a total of 16,324 slices) (Supplementary S4).

For model validation, two test datasets were prepared at the same center (Supplementary S4). From October 2020 to July 2021, out of 821 examinations, 160 examinations (11,514 slices) were identified with grade-1 motion artifacts (no artifacts) and were used for motion artifact simulation. This was achieved by adding phase error components to the Fourier Transform of the magnitude-only images, following a commonly used method [18]. These 160 simulated examinations (11,514 slices) were combined with the paired original MR data as the ground truth to create a paired test dataset. Furthermore, from August 2021 to November 2021, an additional 474 consecutively obtained examinations (33,875 slices) were designated as an unpaired test dataset.

### 2.5. Training, Performance Comparisons, and Ablation Study

The total number of trainable parameters in the DR-CycleGAN network amounts to 39 million. Before training, we performed random cropping for data augmentation on the original images to obtain 128 × 128-pixel images to prevent overfitting issue in the training process [35]. This step can also optimize memory usage and accelerate the training process. All training procedures were carried out on the PyTorch platform using the Adam optimizer. The initial learning rate was set to 0.0001 and exponential decay was applied over 10,000 iterations. Training was conducted on a computer system equipped with an NVIDIA Geforce GTX 2080Ti with 11GB GPU memory and an Intel Core CPU i7-8700 3.7GHz. We compared the performance of our models with a state-of-the-art unsupervised network, Cycle-MedGAN V2.0 [28]. Additionally, we conducted an ablation study on DR-CycleGAN, examining the effects of removing our designed $L_{sum}$ or incorporating an additional artifact encoder for motion-free images. In the paired test dataset, we evaluated and compared the motion-correction accuracies of the technique using the widely used structural similarity index (SSIM) and peak signal-to-noise ratio (PSNR) metrics, as ground truth data were available [36,37]. Furthermore, we compared the motion artifact grades before and after motion correction in both the paired and unpaired test datasets. The flowchart depicting the entire study is presented in Figure 4.

### 2.6. Statistical Analysis

The paired *t*-tests were performed in SPSS software (version 26; IBM) to compare the semi-quantitative and qualitative data. Statistical significance was defined at a *p* value < 0.01 level (two-tailed).

## 3. Results

### 3.1. Evaluation of Paired Simulated Test Dataset

In this section, we assess the performance of DR-CycleGAN in comparison to Cycle-MedGAN V2.0 and a control group consisting of simulated corrupted image data using a paired simulated test dataset.

The evaluation reveals significant differences among the different networks. DR-CycleGAN outperforms both Cycle-MedGAN V2.0 and the control group in terms of SSIM and PSNR, with notably higher values (0.89 ± 0.07 vs. 0.84 ± 0.09/0.81 ± 0.11 and 32.88 ± 2.11 vs. 30.81 ± 2.64/30.13 ± 3.81, respectively; *p* < 0.001 for each comparison). These results indicate that DR-CycleGAN achieved a better signal-to-noise ratio (a higher PSNR) and a closer resemblance to the ground truth (a higher SSIM), signifying better image quality. In contrast, Cycle-MedGAN V2.0 exhibits the lowest SSIM and PSNR values, suggesting suboptimal image quality compared to all other networks.

Additionally, when assessing motion artifact grades, DR-CycleGAN consistently scores lower (2.7 ± 0.7) than both Cycle-MedGAN V2.0 (3.0 ± 0.9) and the control group (4.0 ± 0.8), indicating a superior ability to reduce motion artifacts.

To further visualize these findings, Figure 5 demonstrates the preservation of anatomical details in DR-CycleGAN’s results compared to other networks. For detailed numerical results, please refer to Table 1.

### 3.2. Evaluation of Unpaired Test Dataset

In this section, we assess the performance of DR-CycleGAN and Cycle-MedGAN V2.0 in the context of an unpaired test dataset, focusing on motion artifact correction. This evaluation is essential to gauge the networks’ ability to address real-world scenarios where paired data may not be readily available.

Our findings indicate a marked superiority in the motion artifact correction capabilities of DR-CycleGAN. Specifically, DR-CycleGAN achieved a significant reduction in motion artifact grades, decreasing from an average of 2.9 ± 1.3 to an impressive 2.0 ± 0.6 (*p* < 0.001). This notable improvement underscores its effectiveness in mitigating motion artifacts in unpaired datasets. Conversely, Cycle-MedGAN V2.0 exhibited a notably poorer performance in artifact correction when confronted with the unpaired test dataset. It yielded an average motion artifact grade of 2.4 ± 0.9 (*p* < 0.001), indicating its limitations in effectively addressing motion artifacts under these conditions.

It is noteworthy that Cycle-MedGAN V2.0 exhibited an unexpected behavior by introducing various types of noise in images that were originally free of motion artifacts (grade-1). This behavior is illustrated in Figure 6, highlighting a potential concern regarding the introduction of spurious noise. These results emphasize the robustness and applicability of DR-CycleGAN in real-world scenarios where paired data may be limited, while also raising questions regarding the performance and unintended effects of Cycle-MedGAN V2.0 when handling unpaired datasets.

For a comprehensive presentation of numerical results, please refer to Table 2.

### 3.3. Ablation Study

In this section, we conduct an ablation study to investigate the impact of specific components within DR-CycleGAN, specifically the inclusion of $L_{sum}$ and the presence of the artifact encoder for motion-free images. We aim to assess whether these elements contribute significantly to the network’s performance in reducing artifacts and preserving textural details.

Our visual analysis of the results, as illustrated in Supplementary S6 and S7, suggests noticeable improvements when incorporating $L_{sum}$ and excluding the artifact encoder for motion-free images. These improvements are visually apparent in the reduced presence of artifacts and enhanced preservation of textural details.

However, to provide a more comprehensive understanding, we conducted a statistical analysis. Surprisingly, the statistical analysis revealed no significant differences in the key metrics, including SSIM, PSNR, and motion artifact grade, when comparing models with and without $L_{sum}$ or with and without the artifact encoder for motion-free images. While the visual assessment indicated promising trends, the absence of statistically significant differences in these metrics suggests that the contributions of $L_{sum}$ and the artifact encoder for motion-free images may warrant further investigation or fine-tuning to fully leverage their potential.

The detailed statistical results can be found in Table 3.

### 3.4. Inter-Observer Agreement in Semi-Quantitative Motion Artifact Grading

A total of 1603 examinations were assessed and graded by the aforementioned two radiologists, demonstrating excellent inter-observer agreement with an ICC of 0.965. During the test phase, when grading the motion artifacts in simulated image data (160 examinations), the ICC was 0.957. Additionally, a total of 3170 corrected examinations, comprising 1605 examinations from the paired test dataset and 4745 examinations from the unpaired test dataset, were re-graded after motion corrections, yielding an ICC of 0.960. These high ICC scores underscore a strong consensus between the two radiologists in their grading of motion artifacts for all images.

## 4. Discussion

In this study, we introduce a novel unsupervised network called DR-CycleGAN for effectively correcting motion artifacts in arterial-phase images of gadoxetic acid-enhanced liver MRI examinations. Our network design transforms the motion correction task into an image-to-image translation problem. By leveraging corresponding encoders, the motion-corrupted images are disentangled into content and artifact domains. Subsequently, a generator is employed to extract the motion-free image from the obtained content features. The network incorporates cycle-consistency learning within and across domains, enabling autoencoders to obtain reliable feature representations even in the absence of paired images. Our experimental results demonstrate that DR-CycleGAN outperforms a state-of-the-art unsupervised network, Cycle-MedGAN V2.0, in terms of motion artifact correction [28]. In the test dataset containing simulated corrupted images and paired ground-truth images, DR-CycleGAN achieves significantly higher SSIM and PSNR values compared to Cycle-MedGAN V2.0 (0.89 ± 0.07 vs. 0.84 ± 0.09 and 32.88 vs. 30.81 ± 2.64, respectively; *p* < 0.001 for both measures). Furthermore, in the test dataset consisting of authentic MR images with motion artifacts graded from 1 to 5, DR-CycleGAN demonstrates a significant reduction in motion artifact grades from 2.9 ± 1.3 to 2.0 ± 0.6 (*p* < 0.001), surpassing the performance of Cycle-MedGAN V2.0.

Due to the challenges posed by the lack of ground truth data, supervised learning approaches are seldom practical for the motion correction of gadoxetic acid-enhanced MR images [16,17,18,29]. As a result, the current research focus has shifted towards unsupervised approaches. So far, most unsupervised approaches have been built upon the traditional Cycle-GAN framework, such as Cycle-MedGAN V2.0 and our DR-CycleGAN [20,21,22,23,24,25,26]. However, most of the other published methods share a common limitation: blurring artifacts still persist probably because they have not fully exploited the unique characteristics of motion artifacts while preserving the original content information [20,21,22,23,24,25,26]. In contrast, our network, DR-CycleGAN, stands out by disentangling the artifact component from the true content component without significantly affecting the true content information. Our results clearly demonstrate that DR-CycleGAN outperforms previous studies in terms of motion correction, while maintaining the integrity of anatomical details and avoiding the introduction of additional noise.

In addition to incorporating the conventional adversarial domain loss, reconstruction loss, and cycle-consistency loss, we proposed a novel component called the content-consistency loss ($L_{sum}$) to further enhance our network’s performance. This loss leverages a unique design that efficiently incorporates supervised information through the phase encoding direction [18]. To validate the effectiveness of this approach, we conducted an ablation study, and the results confirmed our hypothesis. It was observed that DR-CycleGAN with $L_{sum}$ achieved a superior artifact reduction performance and preserved finer anatomical structures in the images. This underscores the advantage provided by the inclusion of $L_{sum}$ in our network.

In a recent study by Liu et al., they introduced an unsupervised cycle-consistent adversarial network called DUNCAN, which shared a similar hypothesis with our research [26]. They reported a better motion correction performance compared to other unsupervised methods [26]. However, DUNCAN differs from our network in that it incorporates two content encoders and two artifact encoders specifically for motion-free and motion-corrupted images, probably increasing computational demands and training difficulties. In our ablation study, we examined the use of two artifact encoders in DR-CycleGAN. Surprisingly, the addition of this encoder did not yield any improvement in artifact correction. Instead, it slightly worsened the correction and resulted in a loss of textural details. This suggests that the inclusion of an extra artifact encoder for motion-free images is redundant and may even introduce unintended side effects. Therefore, our proposed DR-CycleGAN not only demonstrates superior effectiveness in removing artifacts and preserving anatomical details across different artifact grades, but also boasts a more streamlined design compared to DUNCAN [26].

This study has certain limitations that should be acknowledged. Firstly, our proposed DR-CycleGAN is specifically designed to address motion artifacts during the arterial phase of gadoxetic acid-enhanced liver MRI data, while other phases such as the portal phase and hepatobiliary phase, which are typically motion-free, are not utilized for motion artifact correction. This limitation restricts the applicability of DR-CycleGAN to only the arterial-phase images. Future research should explore the relationships between gadoxetic acid-enhanced images in different phases. After all, adding multi-phase information may provide more valuable features for correcting motion artifacts in any phase and enable the restoration of finer texture details. Secondly, it is important to acknowledge that the performance of DR-CycleGAN is not yet perfect. This may be attributed to the fact that artifact correction training primarily relies on the learning of reconstructed images rather than the original MR signals. In the next phase of our research, we can explore the possibility of incorporating k-space data for modeling and training purposes. This approach holds the potential to further enhance the effectiveness of artifact correction.

## 5. Conclusions

In conclusion, our DR-CycleGAN framework represents a better motion artifact correction for arterial-phase images acquired during gadoxetic acid-enhanced liver MRI examinations. This approach introduces several key innovations that enhance its efficacy and practicality. First and foremost, DR-CycleGAN simplifies the network architecture by reducing the need for unnecessary encoders, streamlining the computational requirements, and making it more accessible for practical applications in clinical settings. Furthermore, our method extends beyond conventional approaches by considering the crucial correlation between the original motion-corrupted images and the resulting motion-corrected images by applying a novel proposed content-consistency loss function. This consideration ensures that the corrected images not only reduce artifacts, but also retain vital anatomical details, thus enhancing diagnostic accuracy. These advancements collectively demonstrate the transformative potential of DR-CycleGAN in medical imaging. By significantly improving image quality and reducing motion artifacts, our approach holds the promise of revolutionizing radiological diagnosis and positively impacting patient care. As we move forward, this work paves the way for further research in the field of medical image processing. It serves as a foundation for the development of advanced techniques that continue to push the boundaries of motion artifact correction.

## Figures and Tables

**Figure 3:**
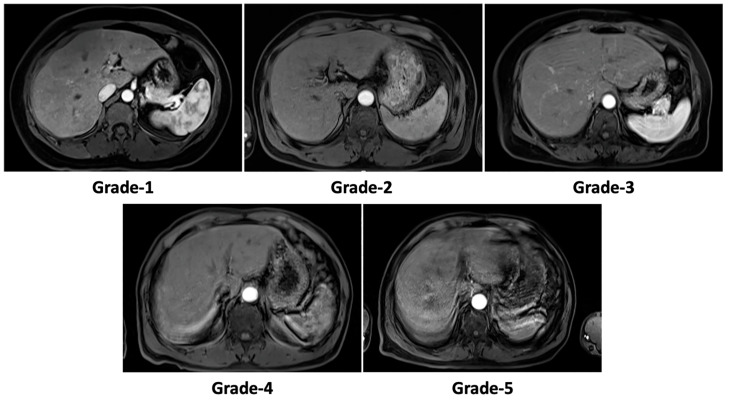
Examples of the five-point Likert scale based on the significance of the artifacts. Notes: grade 1 = no artifact; grade 2 = mild artifacts; grade 3 = moderate artifacts; grade 4 = severe artifacts; grade 5 = non-diagnostic.

**Figure 4:**
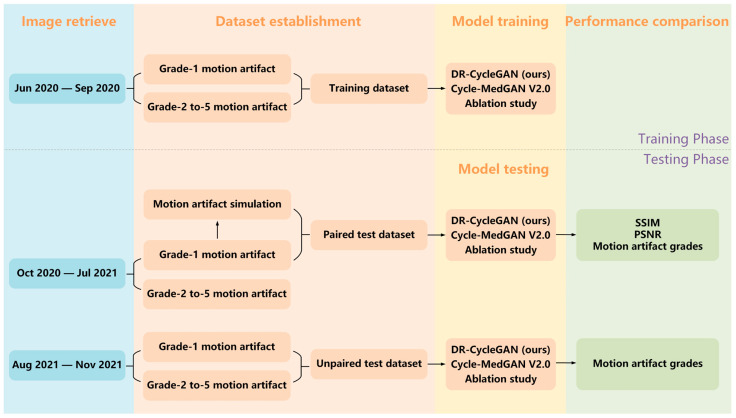
The flowchart of the study.

**Figure 5:**
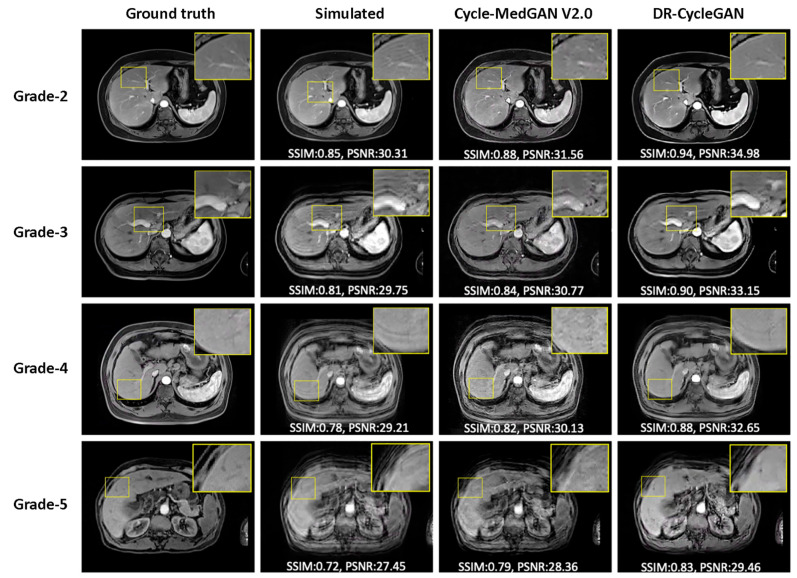
An exemplary illustration of motion artifacts correction results in the paired test dataset.

**Figure 6:**
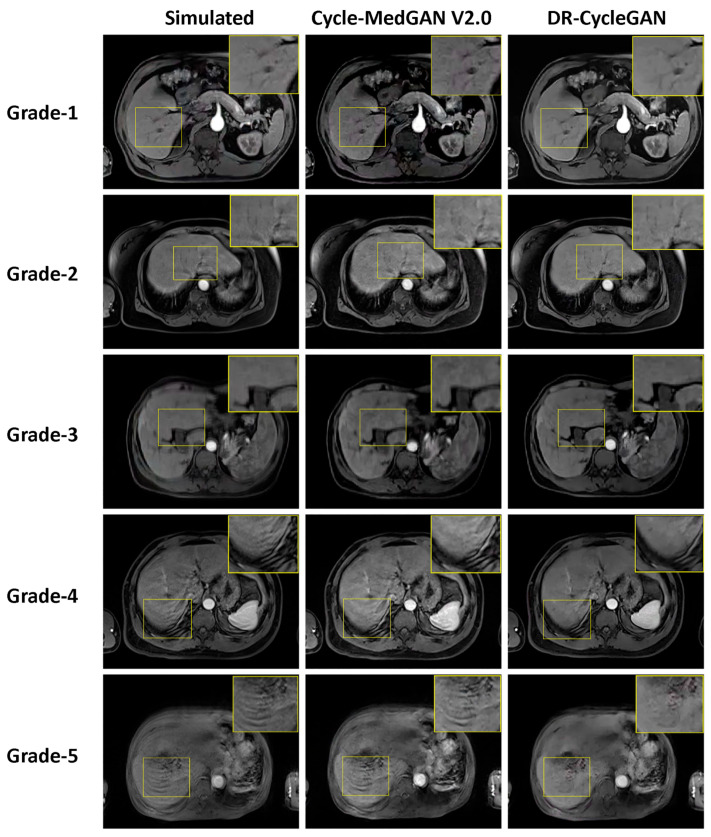
An exemplary illustration of motion artifacts correction results in the unpaired test dataset.

**Table 1:** Comparisons of different models in the paired test dataset (*n* = 160 examinations).

	Mean ± Standard Deviation
**SSIM ^#^**	
*DR-CycleGAN*	0.89 ± 0.07 *
*Cycle-MedGAN V2.0*	0.84 ± 0.09 *
*Simulated corrupted image data*	0.81 ± 0.11 *
**PSNR ^#^**	
*DR-CycleGAN*	32.88 ± 2.11 *
*Cycle-MedGAN V2.0*	30.81 ± 2.64 *
*Simulated corrupted image data*	30.13 ± 3.81 *
**Motion artifact grades**	
*DR-CycleGAN*	2.7 ± 0.7 *
*Cycle-MedGAN V2.0*	3.0 ± 0.9 *
*Simulated corrupted image data*	4.0 ± 0.8 *

^**#**^ SSIM and PSNR were computed as the averages of results across all slices within each examination. * Significances were found when compared with the other two groups (*p* < 0.001).

**Table 2:** Motion artifact grades of different models in the unpaired test dataset (*n* = 474 examinations).

	Mean ± Standard Deviation
**Total (*n* = 474 examinations)**	
*Before correction*	2.9 ± 1.3 *
*DR-CycleGAN*	2.0 ± 0.6 *
*Cycle-MedGAN V2.0*	2.4 ± 0.9 *
**Motion artifact grade-1 (*n* = 60 examinations)**	
*DR-CycleGAN*	1.0 ± 0.0
*Cycle-MedGAN V2.0*	1.0 ± 0.0
**Motion artifact grade-2 (*n* = 157 examinations)**	
*DR-CycleGAN*	1.9 ± 0.3
*Cycle-MedGAN V2.0*	2.0 ± 0.4
**Motion artifact grade-3 (*n* = 110 examinations)**	
*DR-CycleGAN*	2.1 ± 0.5 ^**#**^
*Cycle-MedGAN V2.0*	2.4 ± 0.6
**Motion artifact grade-4 (*n* = 78 examinations)**	
*DR-CycleGAN*	2.4 ± 0.5 ^**#**^
*Cycle-MedGAN V2.0*	3.0 ± 0.7
**Motion artifact grade-5 (*n* = 69 examinations)**	
*DR-CycleGAN*	2.7 ± 0.6 ^**#**^
*Cycle-MedGAN V2.0*	3.8 ± 0.7

* Significances were found when compared with the other two groups (*p* < 0.001). ^**#**^ Significance was found when compared with the other group (*p* < 0.001).

**Table 3:** Results of ablation study.

	Mean ± Standard Deviation
**Paired test dataset (*n* = 160 examinations)**	
**SSIM ^#^**	
*Before correction*	0.81 ± 0.11 *
*DR-CycleGAN*	0.89 ± 0.07
*Without* $L_{sum}$	0.86 ± 0.12
*With artifact encoder for motion-free images*	0.85 ± 0.09
**PSNR ^#^**	
*Before correction*	30.13 ± 3.81 *
*DR-CycleGAN*	32.88 ± 2.11
*Without* $L_{sum}$	32.10 ± 3.04
*With artifact encoder for motion-free images*	32.71 ± 2.47
**Motion artifact grades**	
*Before correction*	4.0 ± 0.8 *
*DR-CycleGAN*	2.6 ± 0.7
*Without* $L_{sum}$	2.8 ± 0.8
*With artifact encoder for motion-free images*	2.7 ± 0.7
**Unpaired test dataset (n = 474 examinations)**	
**Motion artifact grades**	
*Before correction*	2.9 ± 1.3 *
*DR-CycleGAN*	2.0 ± 0.6
*Without* $L_{sum}$	2.2 ± 0.7
*With artifact encoder for motion-free images*	2.2 ± 0.6

^**#**^ SSIM and PSNR were computed as the averages of results across all slices within each examination. * Significances were found when compared with the other three groups (*p* < 0.001).

## Data Availability

The codes are available in the https://github.com/baoqingjia/DR-CycleGAN (accessed on 15 August 2023).

## Supplementary Materials

The following supporting information can be downloaded at: https://www.mdpi.com/article/10.3390/bioengineering10101192/s1, S1: details of the proposed DR-CycleGAN motion correction network; S2: network structures of the proposed encoders and generators; S3: network structures of the proposed discriminators; S4: basic characteristics of datasets; S5: standard scanning protocols of gadoxetic acid-enhanced liver MRI in 1.5T and 2.0T scanners; S6: The comparisons of motion correction by DR-CycleGAN with and without $L_{sum}$ in test datasets; S7: the comparisons of motion correction by DR-CycleGAN with and without artifact encoder for motion-free images.

## References

[1] Park Y.S., Lee C.H., Yoo J.L., Kim I.S., Kiefer B., Woo S.T., Kim K.A., Park C.M. (2016). Hepatic Arterial Phase in Gadoxetic Acid-Enhanced Liver Magnetic Resonance Imaging: Analysis of Respiratory Patterns and Their Effect on Image Quality. Investig. Radiol..

[2] Ichikawa T., Saito K., Yoshioka N., Tanimoto A., Gokan T., Takehara Y., Kamura T., Gabata T., Murakami T., Ito K. (2010). Detection and characterization of focal liver lesions: A Japanese phase III, multicenter comparison between gadoxetic acid disodium-enhanced magnetic resonance imaging and contrast-enhanced computed tomography predominantly in patients with hepatocellular carcinoma and chronic liver disease. Investig. Radiol..

[3] Seo H.J., Kim M.J., Lee J.D., Chung W.S., Kim Y.E. (2011). Gadoxetate disodium-enhanced magnetic resonance imaging versus contrast-enhanced 18F-fluorodeoxyglucose positron emission tomography/computed tomography for the detection of colorectal liver metastases. Investig. Radiol..

[4] Zhuo L.Y., Xing L.H., Ma X., Zhang Y., Ma Z.P., Yin X.P., Wang J.N. (2013). “Nondefect” of arterial enhancing rim on hepatobiliary phase in 3.0-T gadolinium-ethoxybenzyl-diethylenetriamine pentaacetic acid-enhanced liver magnetic resonance imaging: Distinguishing hepatic abscess from metastasis. J. Comput. Assist. Tomogr..

[5] Kim A., Lee C.H., Kim B.H., Lee J., Choi J.W., Park Y.S., Kim K.A., Park C.M. (2012). Gadoxetic acid-enhanced 3.0T MRI for the evaluation of hepatic metastasis from colorectal cancer: Metastasis is not always seen as a “defect” on the hepatobiliary phase. Eur. J. Radiol..

[6] Park Y.S., Lee C.H., Kim B.H., Lee J., Choi J.W., Kim K.A., Ahn J.H., Park C.M. (2013). Using Gd-EOB-DTPA-enhanced 3-T MRI for the differentiation of infiltrative hepatocellular carcinoma and focal confluent fibrosis in liver cirrhosis. Magn. Reason. Imaging.

[7] Zhuo J., Gullapalli R.P. (2006). AAPM/RSNA physics tutorial for residents: MR artifacts, safety, and quality control. Radiographics.

[8] Ikram N.S., Yee J., Weinstein S., Yeh B.M., Corvera C.U., Monto A., Hope T.A. (2017). Multiple arterial phase MRI of arterial hypervascular hepatic lesions: Improved arterial phase capture and lesion enhancement. Abdom. Radiol..

[9] Rimola J., Sapena V., Brancatelli G., Darnell A., Forzenigo L., Mähringer-Kunz A., Paisant A., Renzulli M., Schima W., Terraz S. (2022). Reliability of extracellular contrast versus gadoxetic acid in assessing small liver lesions using liver imaging reporting and data system v.2018 and European association for the study of the liver criteria. Hepatology.

[10] Davenport M.S., Viglianti B.L., Al-Hawary M.M., Caoili E.M., Kaza R.K., Liu P.S., Maturen K.E., Chenevert T.L., Hussain H.K. (2013). Comparison of acute transient dyspnea after intravenous administration of gadoxetate disodium and gadobenate dimeglumine: Effect on arterial phase image quality. Radiology.

[11] Davenport M.S., Caoili E.M., Kaza R.K., Hussain H.K. (2014). Matched within-patient cohort study of transient arterial phase respiratory motion-related artifact in MR imaging of the liver: Gadoxetate disodium versus gadobenate dimeglumine. Radiology.

[12] Well L., Rausch V.H., Adam G., Henes F.O., Bannas P. (2017). Transient Severe Motion Artifact Related to Gadoxetate Disodium-Enhanced Liver MRI: Frequency and Risk Evaluation at a German Institution. Rofo.

[13] Motosugi U., Bannas P., Bookwalter C.A., Sano K., Reeder S.B. (2016). An Investigation of Transient Severe Motion Related to Gadoxetic Acid-enhanced MR Imaging. Radiology.

[14] Inoue Y., Hata H., Nakajima A., Iwadate Y., Ogasawara G., Matsunaga K. (2014). Optimal techniques for magnetic resonance imaging of the liver using a respiratory navigator-gated three-dimensional spoiled gradient-recalled echo sequence. Magn. Reason. Imaging.

[15] Zaitsev M., Maclaren J., Herbst M. (2015). Motion artifacts in MRI: A complex problem with many partial solutions. J. Magn. Reason. Imaging.

[16] Oksuz I., Clough J., Bustin A., Cruz G., Prieto C., Botnar R., Rueckert D., Schnabel J.A., King A.P. (2018). Cardiac MR motion artefact correction from k-space using deep learning-based reconstruction. Proceedings of the 1st Workshop on Machine Learning for Medical Image Reconstruction (MLMIR) held as part of the 21st Conference on Medical Image Computing and Computer Assisted Intervention (MICCAI).

[17] Armanious K., Jiang C., Fischer M., Küstner T., Hepp T., Nikolaou K., Gatidis S., Yang B. (2020). MedGAN: Medical image translation using GANs. Comput. Med. Imaging Graph..

[18] Tamada D., Kromrey M.L., Ichikawa S., Onishi H., Motosugi U. (2020). Motion Artifact Reduction Using a Convolutional Neural Network for Dynamic Contrast Enhanced MR Imaging of the Liver. Magn. Reason. Med. Sci..

[19] Armanious K., Jiang C., Abdulatif S., Küstner T., Gatidis S., Yang B. Unsupervised medical image translation using Cycle-MedGAN. Proceedings of the 27th European Signal Processing Conference (EUSIPCO).

[20] Zhu J.Y., Park T., Isola P., Efros A.A. Unpaired image-to-image translation using cycle-consistent adversarial networks. Proceedings of the 16th IEEE International Conference on Computer Vision (ICCV).

[21] Ghodrati V., Bydder M., Ali F., Gao C., Prosper A., Nguyen K.L., Hu P. (2021). Retrospective respiratory motion correction in cardiac cine MRI reconstruction using adversarial autoencoder and unsupervised learning. NMR Biomed..

[22] Makhzani A., Shlens J., Jaitly N., Goodfellow I., Frey B. (2015). Adversarial autoencoders. arXiv.

[23] Oh G., Lee J.E., Ye J.C. (2020). Unsupervised MR motion artifact deep learning using outlier-rejecting bootstrap aggregation. arXiv.

[24] Breiman L. (1996). Bagging predictors. Mach. Learn.

[25] Chung H., Kim J., Yoon J.H., Lee J.M., Ye J.C. (2021). Simultaneous super-resolution and motion artifact removal in diffusion-weighted MRI using unsupervised deep learning. arXiv.

[26] Liu S., Thung K.-H., Qu L., Lin W., Shen D., Yap P.-T. (2021). Learning MRI artefact removal with unpaired data. Nat. Mach. Intell..

[27] Goodfellow I., Pouget-Abadie J., Mirza M., Xu B., Warde-Farley D., Ozair S., Courville A., Bengio Y. (2020). Generative adversarial networks. Commun. ACM.

[28] Armanious K., Tanwar A., Abdulatif S., Küstner T., Gatidis S., Yang B. Unsupervised adversarial correction of rigid MR motion artifacts. In Proceedings IEEE 17th International Symposium on Biomedical Imaging (ISBI).

[29] Bao Q., Chen Y., Bai C., Li P., Liu K., Li Z., Zhang Z., Wang J., Liu C. (2022). Retrospective motion correction for preclinical/clinical magnetic resonance imaging based on a conditional generative adversarial network with entropy loss. NMR Biomed..

[30] Bai C., Liu K., Chen S., Li Z., Xie W., Bao Q., Liu C. (2022). Dual-domain unsupervised network for removing motion artifact related to Gadoxetic acid-enhanced MRI. J. Phys. Conf. Ser. IOP Publ..

[31] Du W., Chen H., Yang H. Learning invariant representation for unsupervised image restoration. Proceedings of the 2020 IEEE/CVF Conference on Computer Vision and Pattern Recognition (CVPR).

[32] Pietryga J.A., Burke L.M., Marin D., Jaffe T.A., Bashir M.R. (2014). Respiratory motion artifact affecting hepatic arterial phase imaging with gadoxetate disodium: Examination recovery with a multiple arterial phase acquisition. Radiology.

[33] ACR ACOR Liver Imaging Reporting and Data System Version 2018. ACR Web Site..

[34] Yang H., Han P., Huang M., Yue X., Wu L., Li X., Fan W., Li Q., Ma G., Lei P. (2022). The role of gadoxetic acid-enhanced MRI features for predicting microvascular invasion in patients with hepatocellular carcinoma. Abdom. Radiol..

[35] Takahashi R., Matsubara T., Uehara K. (2019). Data augmentation using random image cropping and patching for deep CNNs. IEEE Trans. Circuits Syst. Video Technol..

[36] Wang Z., Bovik A.C., Sheikh H.R., Simoncelli E.P. (2004). Image quality assessment: From error visibility to structural similarity. IEEE Trans. Image Process.

[37] Poobathy D., Chezian R.M. (2014). Edge Detection Operators: Peak Signal to Noise Ratio Based Comparison. Int. J. Image Graph. Signal Process..

