# Supplementary material for: Correction of Arterial-Phase Motion Artifacts in Gadoxetic Acid-Enhanced Liver MRI Using an Innovative Unsupervised Network
Source: Bioengineering (Basel). 2023 Oct 13;10(10):1192. doi: 10.3390/bioengineering10101192 (PMC10604307; doi:10.3390/bioengineering10101192)
Supplement: Supplementary file 1 [file bioengineering-10-01192-s001.zip › bioengineering-2588358-supplementary.pdf]

bioengineering

MDPI

*Article*

# Correction of Arterial-Phase Motion Artifacts in Gadoxetic Acid-Enhanced Liver MRI Using an Innovative Unsupervised Network

**Feng Pan [1,†], Qianqian Fan [1,†], Han Xie [2], Chongxin Bai [3], Zhi Zhang [2], Hebing Chen [1], Lian Yang [1], Xin Zhou [2,4,5], Qingjia Bao [2,*] and Chaoyang Liu [2,4,5,*]**

1 Department of Radiology, Union Hospital, Tongji Medical College, Huazhong University of Science and Technology, Wuhan 430022, China; uh_fengpan@hust.edu.cn (F.P.); qianqianfan@hust.edu.cn (Q.F.); hebing1027898473@126.com (H.C.); yanglian@hust.edu.cn (L.Y.)

2 State Key Laboratory of Magnetic Resonance and Atomic and Molecular Physics, Innovation Academy for Precision Measurement Science and Technology, Chinese Academy of Sciences, Wuhan 430071, China; yingxhgo@gmail.com (H.X.); zhangzhi@apm.ac.cn (Z.Z.); xinzhou@apm.ac.cn (X.Z.)

3 School of Information Engineering, Wuhan University of Technology, Wuhan 430070, China; bcx907372720@foxmail.com

4 University of Chinese Academy of Sciences, Beijing 100864, China

5 Optics Valley Laboratory, Wuhan 430074, China

† Co-first authors

* Correspondence: baoqingjiahaoba@gmail.com (Q.B.); chyliu@wipm.ac.cn (C.L.)

## Electronic Supplementary Material

### S1. Details of the proposed DR-CycleGAN motion correction network

In the training stage, the inputs of DR-CycleGAN are the unpaired motion-free and motion-corrupted images. We assume that the task of motion correction can be considered the image translation problem. Given $x_c \in C$ denotes the training sample (motion-corrupted image) in the motion-corrupted domain ($C$ domain), $x_f \in F$ denotes the training sample (motion-free image) in the motion-free domain ($F$ domain). Since training the proposed DR-CycleGAN does not need paired training data, $x_c$ and $x_f$ are randomly sampled from the unpaired dataset and probably belong to different contents. With inverse translation mapping $T_{c\to f}$: $x_c \to \hat{x}_f$, we translate the motion-corrupted image $x_c$ to the motion-free image $\hat{x}_f$ without aligned image pairs. As this translation mapping ($T_{c\to f}$: $x_c \to \hat{x}_f$) is highly under-constrained, the forward mapping $T_{f\to c}$: $\hat{x}_f \to x_c^{cyc}$ is also designed to translate the generated motion-free image $\hat{x}_f$ back to the original image denoted as $x_c^{cyc}$ with cycle-consistency constraints, following the idea of Cycle-GAN. Specifically, a disentangled representation is introduced in the translation module. The motion-corrupted image can be disentangled into the artifact features $A$ and the content features $C$, and the artifacts feature and the image context feature can be jointly input into the generators to implement paired forward and backward mapping (i.e., motion corruption and its inverse process). Moreover, a novel content loss function based on the MRI physical prior information is proposed. Below, the translation modules and the content consistency loss are described in detail:

*(1) Translation with disentangled representation*

As described above, we combine the disentangled representation within DR-CycleGAN to translate the motion-corrupted images to motion-free images. The network has six translation mappings between the motion-corrupted and motion-free image translation: $T_{c\to f}$: $x_c \to \hat{x}_f$, $T_{f\to c}$: $y_f \to \hat{y}_c$, $T_{c\to c}$: $x_c \to x_c^{rec}$, $T_{f\to f}$: $y_f \to y_f^{rec}$, $T_{f\to c}$: $\hat{x}_f \to x_c^{cyc}$, and $T_{c\to f}$: $\hat{y}_c \to y_f^{cyc}$, in which $x_c$, $\hat{y}_c$, $x_c^{rec}$, and $x_c^{cyc}$ are motion-corrupted images and $y_f$, $\hat{x}_f$, $y_f^{rec}$, and $y_f^{cyc}$ are motion-free images. The $T_{c\to f}$: $x_c \to \hat{x}_f$ and $T_{c\to f}$: $\hat{y}_c \to y_f^{cyc}$ could be

considered inverse translation mappings, where the motion-corrupted image $x_c$ and $\hat{y}_c$ are reconstructed into the motion-free ones $\hat{x}_f$ and $y_f^{cyc}$. On the contrary, the $T_{f\to c}: y_f \to \hat{y}_c$ and $T_{f\to c}: \hat{x}_f \to x_c^{cyc}$ could be considered as forward translation mappings, which translated the motion-free images $y_f$ and $\hat{x}_f$ to the motion-corrupted images $\hat{y}_c$ and $x_c^{cyc}$.

In summary, the detailed process of the translation with disentangled representation, which consists of six components: (1) content encoder ($E_c$); (2) artifact encoder ($E_a$); (3) motion-free image generator ($G_f$); (4) motion-corrupted image generator ($G_c$); (5) motion-free image discriminator ($D_f$); and (6) motion-corrupted image discriminator ($D_c$). The content encoder $E_c$ is used to extract the content features from the motion-free and the motion-corrupted images. For the inverse mapping $T_c$: $x_c \to \hat{x}_f$, the motion-corrupted image $x_c$ is disentangled to obtain the content features $C$ and artifact features $A$ by the content encoder $E_c$ and the artifact encoder $E_a$, respectively. Then, the content features $C$ are decoded by $G_f$ to generate the motion-free image $\hat{x}_f = G_f(E_c(x_c))$. For the forward mapping $T_f: y_f \to \hat{y}_c$, the motion-free image $y_f$ is encoded by $E_c$ to get the content features $C$, and combined with the artifact features $A$. Then, $G_c$ is used to generate the motion-corrupted image $\hat{y}_c = G_c\big(E_c(y_f), E_a(x_c)\big)$. Likewise, the generated motion-free image $\hat{x}_f$ is transformed into a motion-corrupted image $x_c^{cyc} = G_c\big(E_c(\hat{x}_f), E_a(\hat{y}_c)\big)$, and then $G_f$ reconstructs a new motion-free image $y_f^{cyc} = G_f(E_c(\hat{y}_c))$. The discriminator $D_f$ is used to differentiate the reconstructed motion-free image $\hat{x}_f$ from the input motion-free image $y_f$, and the discriminator $D_c$ learns to discriminate the generated motion-corrupted image $\hat{y}_c$ from the input real image $x_c$.

*(2) Content consistency loss ($L_{sum}$)*

The novel *content consistency loss* $L_{sum}$ is proposed based on the MRI physical principle. By calculating the ratio of the summation of the pixel values along each column (the phase-encoding direction) in the motion-free image $y_1$, the motion-corrupted image $\tilde{y}_1$, and the motion-free image $y_2$ at the other slice, we can find that the content features of $y_1$ and $\tilde{y}_1$ at the same slice are much closer than $y_2$ at the other slice. This is because of the presence of motion artifacts caused by breath-holding failures which are always along the column direction (phase-coding direction), resulting in a wave shift along the phase-encoding direction without altering the total signal intensity along each column remaining between the paired motion-free and motion-corrupted images. Thus, we can define a novel *content consistency loss* ($L_{sum}$) to penalize the content discrepancy between the original and translated images, as mentioned in the manuscript, to ensure that the motion-corrected images neither suffer from a loss of anatomical details nor introduce non-existent information.

*(3) Other loss functions*

Besides the content consistency loss $L_{sum}$, the other loss functions are described in detail here:

*a.* The first term $L_{adv}$ is the adversarial domain loss to ensure the generated images are in the corresponding domains. Specifically, the motion-corrupted domain discriminator $D_x$ is employed to distinguish between real and cross-domain-translated motion-corrupted images. Meanwhile, we use the motion-free domain discriminator $D_y$ to differentiate the real and the cross-domain-translated motion-free images. The calculation of $L_{adv}$ follows:

$$L_{adv} = L_{adv}^x + L_{adv}^y \qquad \text{(S.1)}$$

$$L_{adv}^x = E[log D_X(x_c)] + E[\log(1 - D_X(\hat{x}_f)] \qquad \text{(S.2)}$$

$$L_{adv}^y = E\big[log D_Y\big(y_f\big)\big] + E[\log(1 - D_Y(\hat{x}_f)] \qquad \text{(S.3)}$$

where $L_{adv}^x$ and $L_{adv}^y$ represent the motion-corrupted domain adversarial loss and the motion-free domain adversarial loss, respectively, and $E$ denotes the expectation operator.

*b*. The second term $L_{rec}$ is the reconstruction loss, which measures the pixel-wise difference between the input image and its reconstructed counterpart in the within-domain translation:

$$L_{rec} = E(\|x_c - x_c^{rec}\|_1) + E(\|y_f - y_f^{rec}\|_1) \qquad \text{(S.4)}$$

*c*. The cycle-consistency loss $L_{cycle}$ is calculating the difference between the cycle images and the original images:

$$L_{cycle} = E(\|x_c - x_c^{cyc}\|_1) + E(\|y_f - y_f^{cyc}\|_1) \qquad \text{(S.5)}$$

## S2. Network structures of the proposed encoders and generators

The detailed network structures of the proposed encoders and generators are illustrated in Figure 1 below: DR-CycleGAN comprises different encoders and generators. Encoders are further divided into the content encoder ($E_c$) (**a**) and the artifact encoder ($E_a$) (**d**), while the decoders (**c**) consists of a motion-corrupted image generator ($G_c$) and a motion-free image generator ($G_f$). The ResNet structure (**b**) was used in the content encoders and generators.

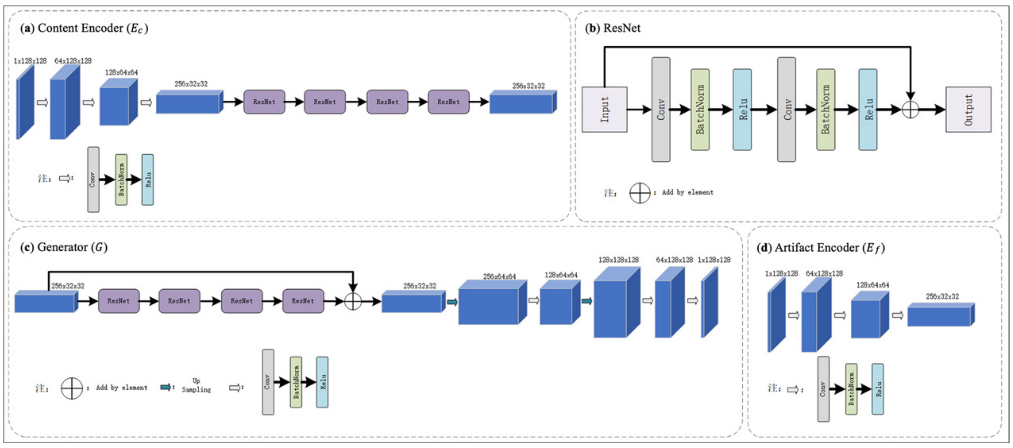


**Figure 1.** Network structures of the proposed encoders and generators.

## S3. Network structures of the proposed discriminators

The detailed network structures of the proposed discriminators are illustrated in Figure 2 below: Within the DR-CycleGAN framework, we have two discriminators ($D_f$ and $D_c$) to differentiate the reconstructed motion-free images ($\hat{x}_f$) and the real motion-free images ($y_f$), as well as between the fake "motion-corrupted" images ($\hat{y}_c$) and the real motion-corrupted images ($x_c$), respectively. To differentiate the images, the input image undergoes a series of operations. It first passes through an average pooling layer and then progresses through five convolutional layers to generate the initial discriminator result. Subsequently, the input image is downsampled and further processed by five additional convolutional layers to obtain the second discriminator result. Lastly, after another downsampling step, the input image undergoes a final set of five convolutional layers to produce the third discriminator result. The discriminator outputs the final result by averaging the three previous results.

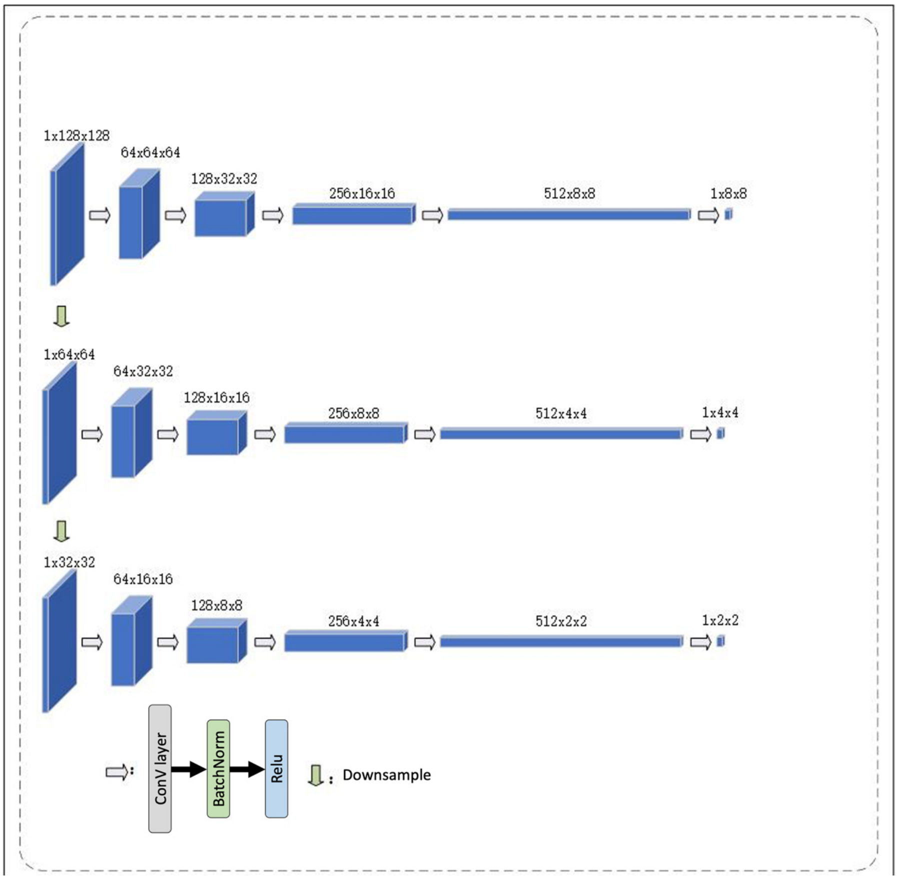


**Figure 2.** Network structures of the proposed discriminators.

**S4.** Basic characteristics of datasets.

| Motion artifact grades | Avanto | Skyra | Total |
|---|---|---|---|
| Motion artifact grades—training dataset | | | |
| Grade-1 | 41 (23.3%) | 17 (12.9%) | 58 (18.8%) |
| Grade-2 | 90 (51.1%) | 67 (50.8%) | 157 (51.0%) |
| Grade-3 | 30 (17.0%) | 31 (23.5%) | 61 (19.8%) |
| Grade-4 | 12 (6.8%) | 14 (10.6%) | 26 (8.4%) |
| Grade-5 | 3 (1.7%) | 3 (2.3%) | 6 (1.9%) |
| Total | 176 (100.0%) | 132 (100.0%) | 308 (100.0%) |
| Motion artifact grades—paired test dataset | | | |
| Grade-1 | 0 (0.0%) | 0 (0.0%) | 0 (0.0%) |
| Grade-2 | 2 (2.3%) | 2 (2.7%) | 4 (2.5%) |
| Grade-3 | 20 (23.0%) | 21 (28.8%) | 41 (25.6%) |
| Grade-4 | 34 (39.1%) | 26 (35.6%) | 60 (37.5%) |
| Grade-5 | 31 (35.6%) | 24 (32.9%) | 55 (34.4%) |
| Total | 87 (100.0%) | 73 (100.0%) | 160 (100.0%) |
| Motion artifact grades—unpaired test dataset | | | |
| Grade-1 | 28 (10.9%) | 32 (14.8%) | 60 (12.7%) |
| Grade-2 | 72 (27.9%) | 85 (39.4%) | 157 (33.1%) |
| Grade-3 | 61 (23.6%) | 49 (22.7%) | 110 (23.2%) |
| Grade-4 | 52 (20.2%) | 26 (12.0%) | 78 (16.5%) |
| Grade-5 | 45 (17.4%) | 24 (11.1%) | 69 (14.6%) |
| Total | 258 (100.0%) | 216 (100.0%) | 474 (100.0%) |

**S5.** Standard scanning protocols of gadoxetic acid-enhanced liver MRI in 1.5T and 2.0T scanners.

| MR Scanning Parameters | 1.5T MR Scanner[1] | 3.0T MR Scanner[2] |
|---|---|---|
| Sequence | 3D-VIBE | 3D-VIBE |
| TR (ms) | 4.74 | 4.50 |
| TE (ms) | 2.38 | 1.29 |
| Flip angle (∘) | 10 | 9 |
| Field of view (mm) | 380 | 380 |
| Base resolution | 320 | 320 |
| Section thickness (mm) | 3 | 3 |
| Contrast[3] dose (mmol/kg) | 0.025 | 0.025 |
| Contrast[3] injection rate (ml/s) | 1.0 | 1.0 |
| **Acquisition phases:** | | |
| Arterial phase (s) | 20-35s | 20-35s |
| Portal vein phase (s) | 60-70s | 60-70s |
| Delayed phase (s) | 180 | 180 |
| HBP (min) | 20min | 20min |

[1]MAGNETOM Avanto, Siemens Healthineers, Germany; [2]MAGNETOM Skyra, Siemens Healthineers, Germany; [3]Primovist®, Bayer Schering Pharma, Berlin, Germany. Abbreviations: MR, magnetic resonance; 3D-VIBE, 3-Dimensional Volumetric Interpolated Breath-hold Examination; TR, repetition time; TE, echo time; HBP, hepatobiliary phase.

## S6. The comparisons of motion correction by DR-CycleGAN with and without $L_{sum}$ in test datasets

The comparisons of motion correction by DR-CycleGAN with and without $L_{sum}$ in test datasets are illustrated in Figure 3 below: **a**, Row 1 and 3 show the different slices in the paired test dataset; row 2 and 4 are the corresponding local enlarged drawings of the area of the red rectangle in the original images above; as a result, DR-CycleGAN with $L_{sum}$ shows a better correction of motion artifacts and reserves more original information due to the effective supervised signals for the translated images. **b**, DR-CycleGAN with $L_{sum}$ also shows a better correction of motion artifact and avoids the fake information formation (referring to the blue rectangle in the model without $L_{sum}$) in the unpaired test dataset.

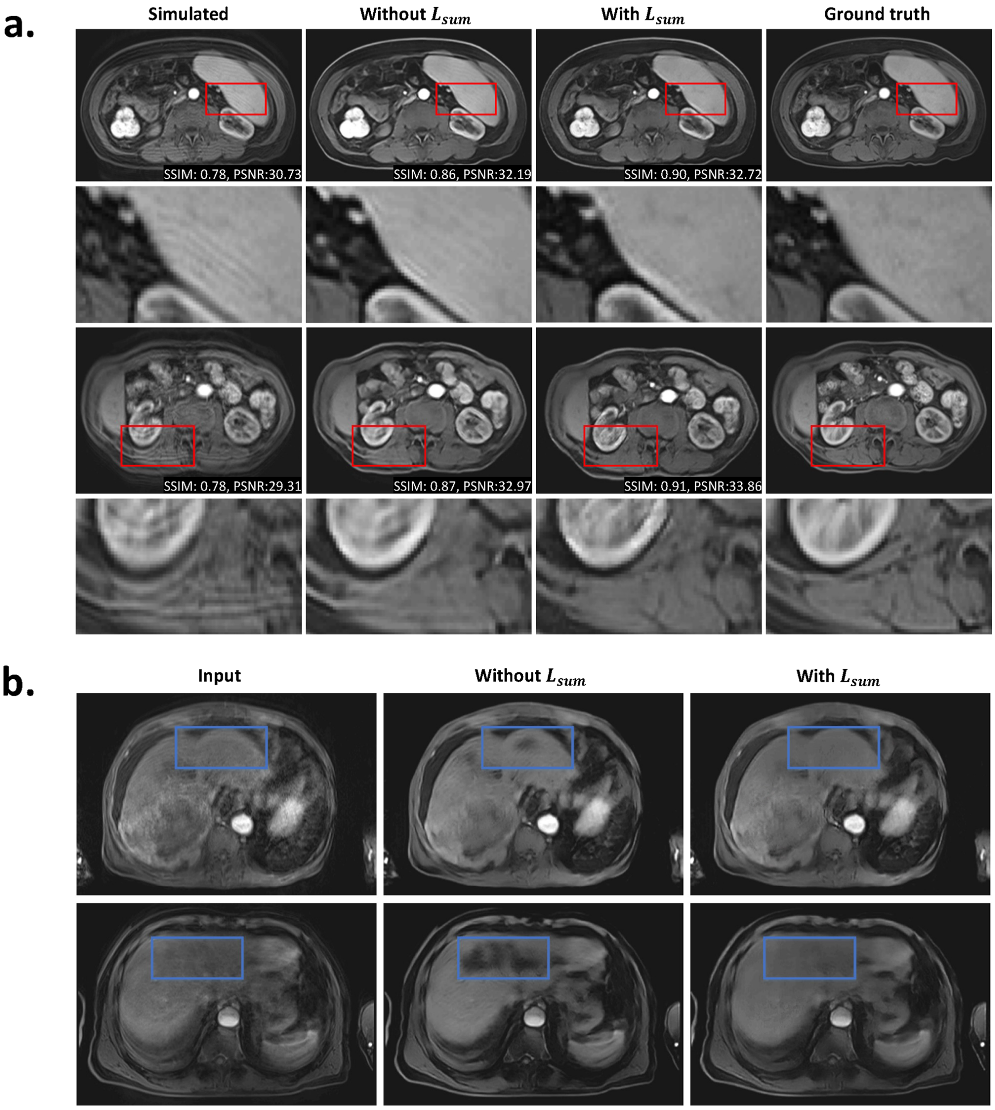


**Figure 3.** The comparisons of motion correction by DR-CycleGAN with and without $L_{sum}$ in test datasets.

## S7. The comparisons of motion correction by DR-CycleGAN with and without artifact encoder for motion-free images

The comparisons of motion correction by DR-CycleGAN with and without artifact encoder for motion-free images are illustrated in Figure 4 below: Row 1 and 3 show the different slices, and row 2 and 4 are the corresponding local enlarged drawings of the area of the red rectangle in the original images. As a result, DR-CycleGAN without an artifact encoder for motion-free images outperforms the network with an artifact encoder for motion-free images, leading to a mildly better image quality with better textural details.

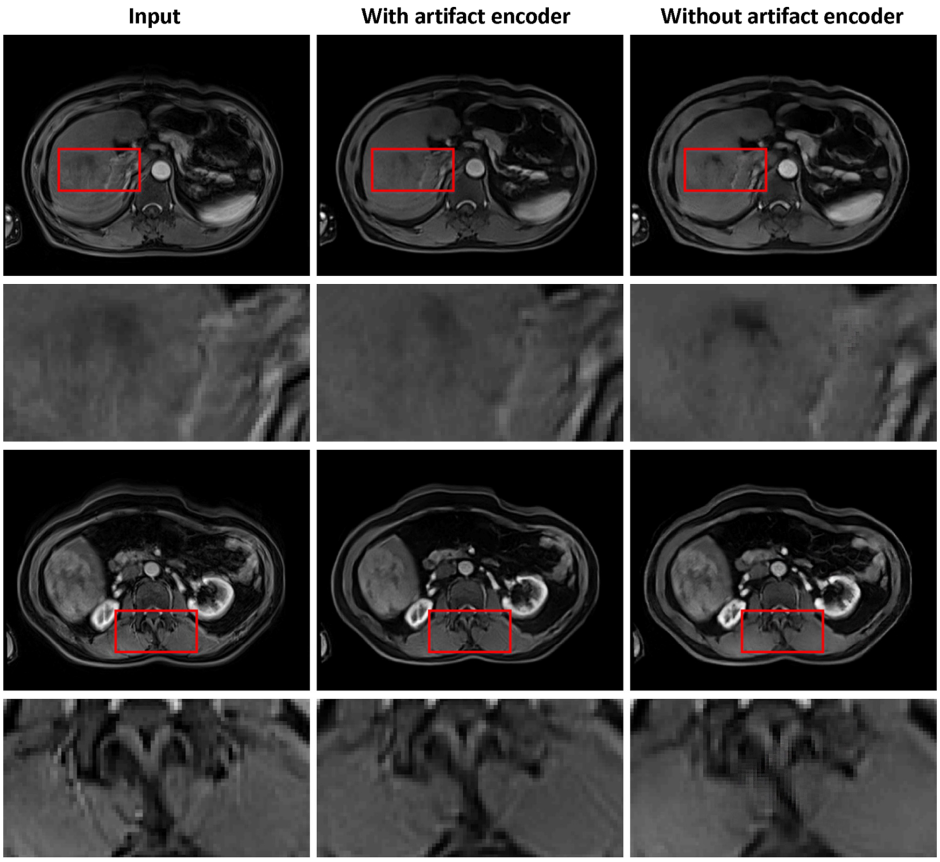


**Figure 4.** The comparisons of motion correction by DR-CycleGAN with and without artifact encoder for motion-free images.
